# Rational Designing of NiO Nanoparticles Anchored with PEG-WO_3_ for Enhanced Water Oxidation Performance

**DOI:** 10.3390/polym17091281

**Published:** 2025-05-07

**Authors:** Mrunal Bhosale, Pritam J. Morankar, Rutuja U. Amate, Chan-Wook Jeon

**Affiliations:** School of Chemical Engineering, Yeungnam University, 280 Daehak-ro, Gyeongsan 712-749, Republic of Korea; mrunal.snst.1@gmail.com (M.B.); pritam.nanoworld@gmail.com (P.J.M.); rutu.nanoworld@gmail.com (R.U.A.)

**Keywords:** electrochemical water splitting, oxygen evolution reaction, hydrogen evolution reaction, electrocatalyst, charge transfer

## Abstract

The electrochemical water splitting method is widely regarded as an efficient and sustainable approach for producing high-purity hydrogen in an environmentally friendly manner. Cost-effective and efficient electrocatalysts are essential for augmenting the electrocatalytic water oxidation reaction. Herein, the PEG-WO_3_-NiO electrocatalyst is acknowledged for attaining efficient oxygen evolution reaction (OER) performances in alkaline conditions. The NiO nanoparticles anchored themselves to the PEG-WO_3_‘s surface and produced an effective interfacial contact between the electrocatalyst materials. Among various compositions, the optimized ratio of the PEG-WO_3_-NiO electrocatalyst exhibits a low overpotential of 349.7 mV at a current density of 10 mA cm^−2^ and a Tafel slope of 71.22 mV dec^−1^ for the OER in 1 M KOH. Additionally, the electrocatalyst demonstrates excellent stability, maintaining its performance even after 5000 cyclic voltammetry (CV) cycles and chronopotentiometry analysis. Given its durability and high electrochemically active surface area, the PEG-WO_3_-NiO electrocatalyst contributes to the advancement of cost-effective and scalable solutions for water oxidation applications.

## 1. Introduction

In the last few years, growing environmental concerns associated with the extensive utilization and reliance on fossil fuels have driven a global shift towards renewable energy sources, including solar, wind, and tidal energy. These green alternatives are increasingly regarded as viable solutions to address the ongoing energy crisis while mitigating pollution and reducing carbon emissions [1,2,3]. Among the various strategies for sustainable energy production, electrochemical water splitting has emerged as a highly promising approach for generating high-purity hydrogen, which serves as an efficient and clean fuel with broad industrial applications [4,5]. Water electrolysis is fundamentally governed by two critical half-reactions, i.e., the hydrogen evolution reaction (HER) and the oxygen evolution reaction (OER). These reactions require highly efficient electrocatalysts to lower the activation energy and enhance the overall energy conversion efficiency. In the field of water splitting, noble metal-based materials have long been recognized as benchmark electrocatalysts due to their exceptional catalytic activity and stability. Specifically, IrO_2_ and RuO_2_ exhibit superior OER activity, while Pt- and Pd-based catalysts are widely regarded for their outstanding HER performance. However, the widespread industrial implementation of these noble metal catalysts is significantly constrained by their high cost and limited natural abundance [6,7,8]. This has encouraged widespread research into developing cost-effective and earth-abundant alternative materials with comparable or superior electrocatalytic performance.

Transition metal-based catalysts, including those derived from nickel, cobalt, and iron, as well as heterostructured and nanostructured materials have demonstrated great potential in addressing these challenges. Tungsten oxide (WO_3_) exhibits a tunable crystalline phase and a highly adaptable structure, allowing for the precise modulation of its catalytic performance through structural engineering and surface electronic modifications [9,10,11]. Conversely, the widespread application of tungsten oxide in electrocatalysis is constrained by several inherent limitations, including its adequate catalytic activity, limited density of active sites, and low intrinsic electronic conductivity [10,12,13,14]. These challenges hinder its overall efficiency and necessitate further advancements in material design and surface engineering to enhance its electrocatalytic performance [10,12]. Various effective strategies have been developed to optimize these characteristics, thus enhancing its electrocatalytic efficiency. Moreover, tungsten oxide-based materials have garnered significant research interest due to their ease of synthesis and remarkable stability in extreme pH conditions. These combined properties position tungsten oxide as a highly promising candidate for water electrocatalysis [9]. Nickel-based electrocatalysts have gained significant attention in alkaline electrochemical systems due to their superior catalytic activity which can be attributed to their high density of active sites and unique electronic and structural properties [15,16,17]. Additionally, nickel exhibits excellent electrochemical stability under alkaline conditions, further contributing to its effectiveness as an electrocatalyst. The integration of nickel compounds into hybrid electrocatalysts offers multiple advantages, leading to significant improvements in overall electrochemical performance. This enhancement arises from the following three key factors: (i) the inherent OER activity of nickel, which accelerates reaction kinetics; (ii) improved charge transfer efficiency; and (iii) the formation of strong interfacial interactions that generate synergistic effects resulting in enhanced structural integrity and catalytic stability [16,18]. Recent developments have recognized NiO nanostructures as highly promising electrocatalysts for the OER. Their advantages include fast electrochemical response, excellent corrosion resistance, natural abundance, cost-effectiveness, improved surface kinetics, and long-term stability [19,20]. For example, in a 1 M KOH electrolyte, zero-dimensional (0D) NiO nanoparticles demonstrated an overpotential of 373 mV at a current density of 10 mA cm^−2^. In contrast, two-dimensional (2D) porous NiO nanoplates exhibited a higher overpotential of 476 mV under identical conditions, indicating the influence of morphological differences on OER performance [21]. The NiO hollow spheres calcined at 300 °C (NiO-300) exhibited notable bifunctional electrocatalytic activity for both the OER and HER in 1 M KOH. NiO-300 required an overpotential of 424 mV for HER and 370 mV for OER to achieve a current density of 10 mA cm^−2^, highlighting its efficiency as a dual-functional electrocatalyst [22]. The Ag_2_O–NiO nanostructures exhibit remarkable electrocatalytic performance, requiring an overpotential of 140 mV for the HER and 430 mV for the OER at 10 mA cm^−2^. Additionally, the Ag_2_O/NiO composite demonstrates outstanding long-term stability maintaining efficient OER and HER activity over an extended operational period [23].

In this study, we synthesized PEG-WO_3_-NiO nanostructured electrocatalysts and demonstrated their enhanced electrocatalytic performance for the alkaline OER. The incorporation of NiO with PEG-WO_3_ was aimed at boosting the catalytic activity. Spectroscopic analyses confirmed the successful formation of the PEG-WO_3_-NiO nanocomposite, with NiO nanoparticles uniformly anchored on the PEG-WO_3_ surface. Among the various compositions tested, PWNiO_1.5_ exhibited the most favorable electrochemical characteristics, showing a low overpotential of 349.7 mV at a current density of 10 mA cm^−2^ and a Tafel slope of 71.22 mV·dec^−1^. The PEG-WO_3_-NiO catalyst significantly enhances the transfer of active species and improves electrocatalytic performance primarily due to its increased active surface area and synergistic effects. Furthermore, the stability of the electrocatalyst was confirmed through extended cyclic voltammetry and chronopotentiometry measurements.

## 2. Experimental Section

### 2.1. Chemicals

Nickel nitrate hexahydrate (Ni(NO_3_)_2_·6H_2_O), sodium tungstate dihydrate (Na_2_WO_4_·2H_2_O, ≥99%), poly(ethylene glycol) (PEG), polyvinylidene fluoride (PVDF), and N-methyl-2-pyrrolidone (NMP, ≥99%) were procured from Sigma-Aldrich, St. Louis, MO, USA. Hydrochloric acid (HCl, extra pure) and ethanol (EtOH, C_2_H_5_OH, 94.5%) were sourced from Duksan Chemicals, Gyeonggi-do, Republic of Korea. Potassium hydroxide (KOH, >85%) was supplied by Daejung Chemicals & Metals, Gyeonggi-do, Republic of Korea. Acetylene black (99.9+%) was obtained from Thermo Scientific, Seoul, Republic of Korea. The carbon cloth utilized in this study was acquired from NARA Cell-Tech Corporation, Seoul, Republic of Korea. All chemicals were used without further purification, and deionized (DI) water was exclusively employed in all experimental procedures to ensure consistency and reliability in the study.

### 2.2. Preparation of PEG-WO_3_-NiO

**Figure 1:**
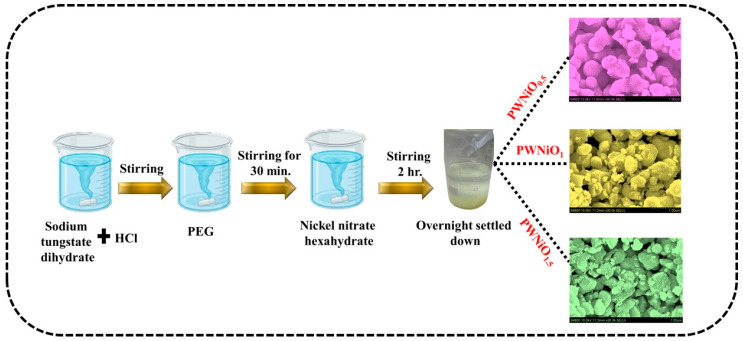
Schematic illustration of synthesis of the PEG-WO_3_-NiO nanocomposites.

The synthesis of the PEG-WO_3_-NiO nanostructures was conducted via a hydrochloric acid-assisted precipitation technique, as depicted in Figure 1. Initially, Na_2_WO_4_·2H_2_O was dissolved in 50 mL of DI water under continuous stirring for 30 min to achieve complete dissolution. Subsequently, diluted HCl was incrementally introduced into the homogeneous solution until the pH reached approximately 2. Following pH adjustment, 3% PEG was incorporated and stirred for an additional 30 min to ensure complete dissolution. Once the PEG was fully dissolved, varying concentrations (0.5, 1, and 1.5%) of Ni(NO_3_)_2_·6H_2_O were introduced into the solution. The mixture underwent vigorous stirring for a further 2 h before being left undisturbed overnight to facilitate precipitation. After the precipitation process, the supernatant was carefully decanted, and the resulting precipitate was thoroughly washed and subsequently dried at 120 °C for 3 h. The dried material was then subjected to thermal annealing at 450 °C for 3 h, followed by grinding using an agate mortar and pestle to obtain the final nanostructured product. The PEG-WO_3_-NiO composite was made with three different concentrations of Ni(NO_3_)_2_.6H_2_O (0.5, 1, and 1.5%), designated as PWNiO_0.5_, PWNiO_1_, and PWNiO_1.5_, respectively.

### 2.3. Material Characterization

The structural properties of the synthesized nanomaterials were examined using an X-ray diffractometer (X’Pert Pro, PAN Analytical, Almelo, The Netherlands) equipped with a Cu Kα radiation source. Raman spectroscopy was carried out with an XploRA Plus system (HORIBA Jobin Yvon S.A.S, Paris, France) to analyze the vibrational characteristics. The surface chemical composition and oxidation states were investigated through X-ray photoelectron spectroscopy (XPS) using a Thermo Scientific K-Alpha surface, Cheshire, UK analysis system. Additionally, the surface morphology, particle size distribution, elemental composition, and elemental mapping were evaluated using scanning electron microscopy (SEM, HITACHI S-4800, Tokyo, Japan) integrated with an energy-dispersive X-ray (EDX) analysis system and high-resolution transmission electron microscopy (HRTEM, Tecnai F21, FEI Company, Tokyo, Japan).

### 2.4. Electrochemical Analysis

A Biologic Instrument WBCS3000 battery cycler, Gières, France was used to perform electrochemical measurements. A three-electrode arrangement was employed for comprehensive analysis. Prior to coating the electrocatalyst onto carbon cloth (CC) the substrate underwent a pre-treatment process involving sequential sonication in 1 M HCl, DI water, and ethanol for 20 min each, followed by drying at 70 °C overnight. The electrocatalyst paste was formulated with an 80:10:10 weight ratio of active material, PVDF, and acetylene black using NMP as the solvent. The prepared slurry was uniformly coated onto pre-treated 1 × 1 cm^2^ carbon cloth and dried at 60 °C overnight to ensure proper adhesion. For electrochemical characterization, the coated carbon cloth functioned as the working electrode, while a Hg/HgO electrode and a platinum plate functioned as the reference and counter electrodes, respectively. All electrochemical measurements were carried out in a N_2_-saturated 1 M KOH electrolyte solution. Cyclic voltammetry (CV) was analyzed in the non-Faradaic region within a potential range of 0.1 to 0.2 V at varying scan rates of 5, 10, 15, 20, and 25 mV s^−1^. The ECSA was subsequently calculated using the following equation [24]:

(1) $$ECSA=\frac{C_{dl}}{C_{s}}$$

where C_dl_ signifies the electrochemical double-layer capacitance, while C_s_ indicates the specific capacitance of a planar surface in a 1 M KOH electrolyte, which is reported to be 0.040 mF cm^−2^ [25,26]. To determine the overpotential for the OER, linear sweep voltammetry (LSV) was conducted at a scan rate of 5 mV s^−1^ within a potential range of 0 to 1 V. All electrochemical measurements were performed using the Hg/HgO reference electrode, and the attained data were converted to the reversible hydrogen electrode (RHE) scale using the Nernst equation, as follows:

E_RHE_ = E_Hg/HgO_ + E°_Hg/HgO_ + 0.0591 × (pH) (2)

where E°_Hg/HgO_ represents the standard electrode potential of the Hg/HgO reference electrode, and the pH of a 1 M KOH solution is approximately 13.9.

LSV was utilized to relate the optimized electrocatalysts stability before and after 5000 cycles of CV study.

## 3. Result and Discussion

**Figure 2:**
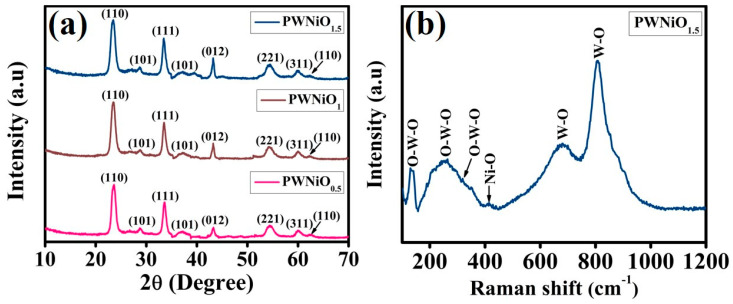
(**a**) XRD spectra of all the composites and (**b**) Raman spectra of the PWNiO_1.5_ composite.

The structural integrity, crystallinity, and phase purity of the synthesized materials were thoroughly examined using X-ray diffraction (XRD) analysis. Figure 2a presents the XRD spectra of PWNiO_0.5_, PWNiO_1_, and PWNiO_1.5_ composites providing critical insights into their phase composition. The diffraction pattern of WO_3_ exhibits distinct peaks at 23.5°, 28.8°, 33.5°, 54.2°, and 60.0° which correspond to the (110), (101), (111), (221), and (311) crystal planes, respectively. These peak positions align well with the standard diffraction data from the JCPDS card No. 01-085-0808, confirming the successful formation of WO_3_ with its characteristic crystalline structure. Additionally, the presence of nickel oxide within the composite is indicated by prominent diffraction peaks observed at 37.2°, 43.2°, and 62.5° which are attributed to the (101), (012), and (110) lattice planes, respectively. The NiO phase exhibits a rhombohedral crystal structure consistent with JCPDS card No. 01-044-1159 [27,28]. A noticeable trend in the XRD patterns reveals that as the concentration of nickel increases, the intensity of the corresponding diffraction peaks also intensifies further validating the successful incorporation of nickel into the composite matrix. These findings confirm the successful formation of the PEG-WO_3_-Ni nanocomposite with well-defined crystallinity and phase purity. The structural characteristics as revealed by XRD support the potential applicability of this composite in electrocatalytic processes where well-crystallized phases contribute to enhanced catalytic activity and stability. To examine the molecular interactions within the PEG-WO_3_-NiO nanocomposites, Raman scattering analysis was performed, as illustrated in Figure 2b. The PWNiO_1.5_ nanocomposite exhibited distinct Raman peaks at 129, 264, 318, 414, 699, and 808 cm^−1^. The peak observed at 129 cm^−1^ corresponds to the antisymmetric stretching vibrations of O–W–O bonds [12]. The bending vibrational modes associated with the O–W–O bridging bonds were identified at 264 and 318 cm^−1^ [29,30]. Additionally, the peaks appearing at 699 and 808 cm^−1^ are characteristic of the WO_3_ corresponding to W–O stretching vibrations [12]. Furthermore, the Raman peak detected at 414.3 cm^−1^ is attributed to the Ni–O stretching mode, indicating the presence of NiO species [31,32].

**Figure 3:**
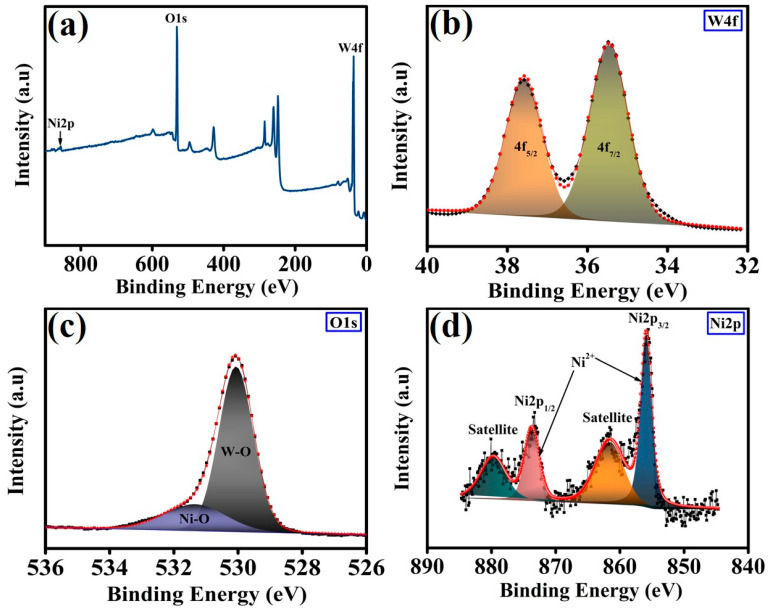
(**a**) High-resolution XPS spectra, (**b**) W4f, (**c**) O1s, and (**d**) Ni2p spectra of PWNiO_1.5_ composite.

The surface chemical composition and valence states of tungsten (W), oxygen (O), and nickel (Ni) in the PWNiO_1.5_ nanocomposite were analyzed using X-ray photoelectron spectroscopy (XPS). The full XPS survey spectrum (Figure 3a) confirms the presence of W, O, and Ni elements within the composite. To further elucidate the chemical states of these elements, the XPS spectra were deconvoluted, and the high-resolution spectra of W4f, O1s, and Ni2p are presented in Figure 3b,c,d, respectively. The deconvoluted W4f spectrum (Figure 3b) exhibits two distinct peaks at binding energies of 35.4 eV and 37.5 eV, corresponding to the 4f_7/2_ and 4f_5/2_ orbitals of W^6+^ in the WO_3_ phase, respectively [33,34,35]. The high-resolution O1s spectrum (Figure 3c) reveals a peak at 530.0 eV that is attributed to W–O bonds in WO_3_ [34]. One additional peak is present at 531.3 eV which shows Ni–O bonding in the PWNiO_1.5_ composite [28,36]. The Ni2p’s high-resolution spectrum (Figure 3d) was deconvoluted into four characteristic peaks. The peak at 855.8 eV corresponds to Ni2p_3/2_, while the satellite peak associated with Ni2p_3/2_ appears at 861.5 eV [23,37]. Another peak at 873.6 eV is assigned to Ni2p_1/2_, with an additional shake-up satellite peak observed at 879.5 eV [23]. These spectral features indicate the presence of a NiO structure, suggesting the partial oxidation of Ni^2+^ species within the composite.

**Figure 4:**
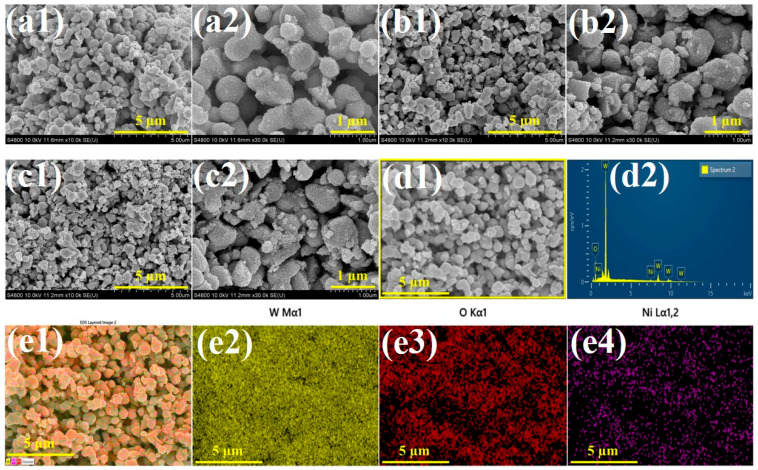
SEM micrograph images of (**a1**,**a2**) PWNiO_0.5_, (**b1**,**b2**) PWNiO_1_, and (**c1**,**c2**) PWNiO_1.5_. (**d1**,**d2**) Energy-dispersive X-ray spectroscopy analysis of PWNiO_1.5_, and (**e1**–**e4**) elemental mapping data of PWNiO_1.5_ electrocatalyst.

The morphological features and elemental configuration of the synthesized electrocatalyst materials were investigated using scanning electron microscopy (SEM) and energy-dispersive X-ray spectroscopy (EDAX). Figure S1 and Figure 4 depict the morphological variations in WO_3_ and PEG-WO_3_-NiO composites with different compositions, respectively. SEM analysis of WO_3_ (Figure S1a,b) reveals an interconnected nanogranular structure with a droplet-like morphology. Upon the incorporation of PEG, a noticeable increase in nanogranular size is observed in the PWNiO_0.5_, PWNiO_1_, and PWNiO_1.5_ composites. The nickel oxide component is present in a nanoparticle-like structure. However, in the PWNiO_0.5_ (Figure 4(a1,a2)) its distribution appears relatively low which may limit its overall catalytic effectiveness. In the case of PWNiO_1_ (Figure 4(b1,b2)) and PWNiO_1.5_ (Figure 4(c1,c2)) an increase in NiO nanoparticle formation is observed with a higher Ni content. Notably, in the PWNiO_1.5_ composite, NiO nanoparticles are well attached to the PEG-WO_3_ matrix, exhibiting a uniform distribution. Elemental composition analysis of the PWNiO_1.5_ composite was performed using EDAX, as presented in Figure 4(d1,d2). The analysis confirms the presence of W, O, and Ni, with their respective weight percentages determined as 75.59%, 18.11%, and 6.31%. To evaluate the uniformity of the elemental distribution, EDAX mapping was conducted as illustrated, in Figure 4(e1–e4). The mapping results indicate a homogeneous dispersion of NiO nanoparticles throughout the PEG-WO_3_ matrix. This uniform distribution suggests that enhanced electrocatalytic performance can be achieved by facilitating effective interaction between the active sites, which may contribute to improved catalytic activity. The morphological and structural characteristics of the synthesized PWNiO_1.5_ composite were further elucidated through high-resolution transmission electron microscopy (HRTEM), as shown in Figure S2a,b. The HRTEM image reveals a densely packed structure composed of PEG assisted WO_3_ nanogranules with uniformly dispersed NiO nanoparticles. The nanogranular morphology of the WO_3_ is evident, forming a constant and interconnected network. The darker contrast spots correspond to the higher electron-dense NiO nanoparticles embedded on the PEG-WO_3_ nanogranulars. The NiO nanoparticles marked with yellow squares are seen to be well-dispersed, suggesting effective confinement within the PEG-WO_3_ framework during synthesis.

**Figure 5:**
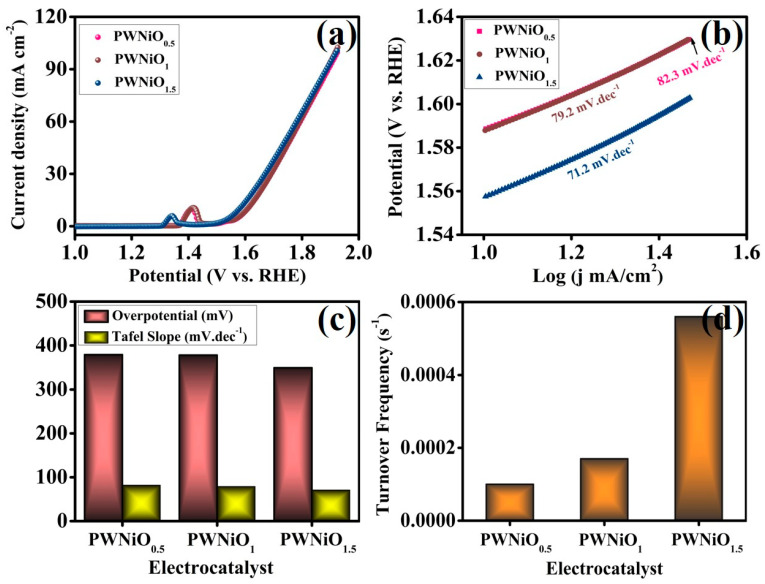
Electrochemical characterizations of electrocatalyst OER performance: (**a**) LSV curves at a 5 mV/s scan rate, (**b**) analogous Tafel slopes, (**c**) assessment of the OER performance concerning overpotential at 10 mA cm^−2^ and Tafel slope, and (**d**) turnover frequency results.

The evaluation of catalytic activity in an alkaline medium is conventionally determined by monitoring oxygen evolution. A key parameter in assessing the efficiency of electrocatalysts is the overpotential at a specified current density, as it directly influences catalytic performance [38]. Consequently, comprehensive electrochemical characterization were conducted to analyze the OER activity. The electrochemical assessments were performed using a three-electrode configuration, where the synthesized materials were deposited onto carbon cloth as the working electrode while Hg/HgO and a platinum plate functioned as the reference and counter electrodes, respectively. Linear sweep voltammetry (LSV) measurements were carried out in a 1 M KOH electrolyte at a scan rate of 5 mV s^−1^ to investigate the electrocatalytic behavior of the PEG-WO_3_-NiO catalyst systems. As illustrated in Figure 5a, the PWNiO_1.5_ electrocatalyst demonstrates superior OER performance, achieving an overpotential of 349.7 mV at a current density of 10 mA cm^−2^. When compared to other electrocatalysts, i.e., PWNiO_0.5_ (379.1 mV) and PWNiO_1_ (378.1 mV), PWNiO_1.5_ exhibits significantly enhanced electrocatalytic activity under identical experimental conditions. This improvement in catalytic efficiency can be attributed to the increasing concentration of Ni within the composite, suggesting a direct correlation between Ni content and performance enhancement. To further investigate the influence of the electrocatalyst composition on reaction kinetics, the Tafel slopes were analyzed, as shown in Figure 5b. The measured Tafel slope values for PWNiO_0.5_, PWNiO_1_, and PWNiO_1.5_ were 82.3, 79.2, and 71.2 mV dec^−1^, respectively. Notably, PWNiO_1.5_ exhibited the lowest Tafel slope, indicating improved charge transfer kinetics, which aligns with its lower overpotential, as depicted in Figure 5c. The Tafel dynamic analysis revealed that the PWNiO_1.5_ electrocatalyst exhibits a Tafel slope of 71.2 mV dec^−1^, signifying that the RDS in the OER is linked to the conversion of Ni–O species into Ni–OOH [39]. The intrinsic electrocatalytic activity of the synthesized samples was further evaluated through the calculation of the turnover frequency (TOF). TOF values were derived from the integrated charge associated with the reduction peak areas at 300 mV fixed potential, as illustrated in Figure S3a–c [40,41]. Among the investigated samples, the PWNiO_1.5_ catalyst exhibits the highest TOF value of 5.6 × 10^−4^ s^−1^, substantially superior those of PWNiO_0.5_ (1.0 × 10^−4^ s^−1^) and PWNiO_1_ (1.7 × 10^−4^ s^−1^), as depicted in Figure 5d. The enhanced TOF of PWNiO_1.5_ reflects a greater density of accessible active sites and a more efficient catalytic turnover, thus confirming its superior OER kinetics. These findings promotes faster oxygen evolution per active site and contribute significantly to the overall electrocatalytic enhancement.

**Figure 6:**
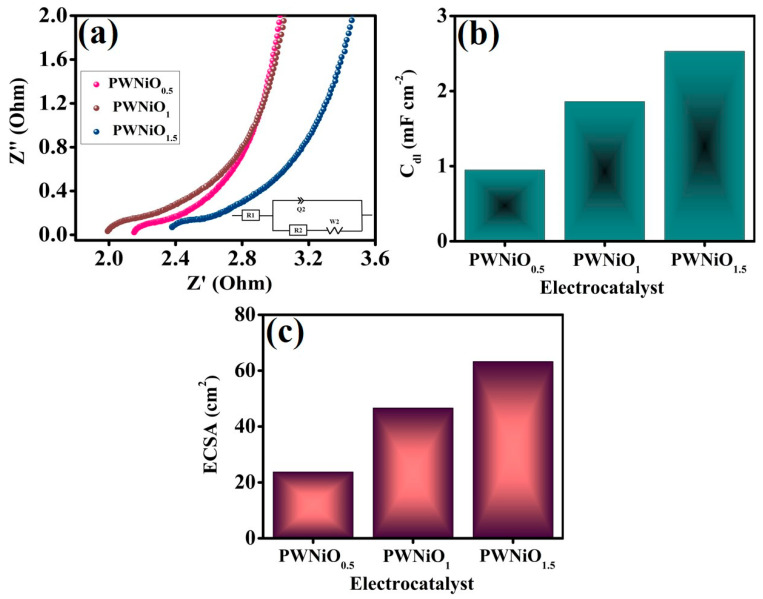
(**a**) EIS spectra, (**b**) C_dl_, and (**c**) ECSA results of all of the electrocatalysts.

Electrochemical impedance spectroscopy (EIS) was utilized to evaluate the charge transfer kinetics involved in OER, as depicted in Figure 6a. To achieve a more precise understanding of the electrochemical characteristics, the EIS data were fitted to equivalent circuit models. The charge transfer resistance (R_ct_) values for PWNiO_0.5_, PWNiO_1_, and PWNiO_1.5_ were determined to be 18.5, 14.7, and 9.7 Ω, respectively. The lower R_ct_ value of PWNiO_1.5_ signifies enhanced charge transport capabilities for OER processes. A reduced R_ct_ reflects an accelerated electron transfer rate at the electrode–electrolyte interface which is crucial for optimizing the overall catalytic activity. Figure S4 presents the cyclic voltammetry (CV) curves for various PEG-WO_3_-NiO ratios at different scan rates along with a corresponding plot of current density versus potential. The CV analysis indicates that an increase in scan rate results in a higher current density highlighting the dependence of electrochemical activity on scan rate variations. To further investigate the correlation between electrocatalytic performance and active sites, the electrochemically active surface area (ECSA) was estimated based on the double-layer capacitance (C_dl_), as depicted in Figure 6b and 2C_dl_ in Figure S5. As shown in Figure 6b, the C_dl_ values for PWNiO_0.5_, PWNiO_1_, and PWNiO_1.5_ were determined to be 0.95, 1.86, and 2.53 mF cm^−2^, respectively. Correspondingly, the calculated ECSA values illustrated in Figure 6c were 23.75, 46.62, and 63.25 cm^2^ for PWNiO_0.5_, PWNiO_1_, and PWNiO_1.5_, respectively. The significant enhancement in ECSA with increasing Ni content suggests that the optimized incorporation of Ni effectively enhances the active sites of V, which play a pivotal role in improving the overall electrochemical performance for the OER [42].

**Figure 7:**
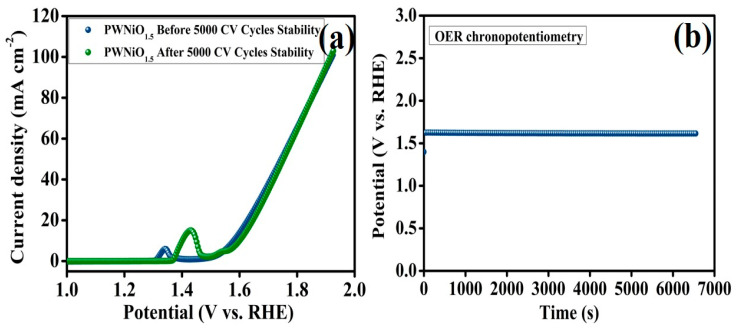
(**a**) LSV curves of PWNiO_1.5_ before and after 5000 CV cycles and (**b**) chronopotentiometry analysis.

The long-term stability of electrocatalysts is a crucial factor in electrochemical performance evaluation, particularly for potential commercial applications. To assess the structural and electrochemical durability of the heterostructured PWNiO_1.5_ electrocatalyst in a 1.0 M KOH electrolyte, cyclic voltammetry (CV) studies and chronopotentiometry measurements were conducted. The CV cycling test was performed at a scan rate of 50 mV s^−1^ in a 1.0 M KOH electrolyte to monitor the durability of the electrocatalyst under prolonged operational conditions. As depicted in Figure 7a, the polarization curve of PWNiO_1.5_ was recorded before and after 5000 CV cycles to analyze its performance retention. After undergoing 5000 cycles, the electrocatalyst maintained an overpotential of 370.3 mV at a current density of 10 mA cm^−2^ which closely matches the initial values obtained before cycling, demonstrating excellent electrochemical stability. Furthermore, a chronopotentiometry experiment was conducted at a current density of 10 mA cm^−2^ over a continuous 7000 s (Figure 7b) to further examine the catalyst’s endurance. The PWNiO_1.5_ electrocatalyst exhibited remarkable stability. However, a slight loss in performance could be attributed to several factors, including the minor leaching of active materials from the electrode’s surface, inadequate diffusion of generated O_2_ away from the reaction interface, or potential structural degradation due to oxidation under high potentials [27,43].

## 4. Conclusions

A PEG-WO_3_-NiO electrocatalyst was successfully synthesized via a co-precipitation technique demonstrating promising potential for OER applications. In the PEG-WO_3_-NiO composite, NiO nanoparticles are well-integrated within the PEG-WO_3_ matrix, forming a homogeneous and uniform dispersion which plays a crucial role in enhancing its electrocatalytic activity. Among the various synthesized compositions, PWNiO_1.5_ exhibited the most outstanding electrocatalytic performance, requiring a relatively low overpotential of 349.7 mV to achieve a current density of 10 mA cm^−2^. This superior activity can be attributed to the synergistic interactions between the NiO nanoparticles and the PEG-WO_3_ structure which enhance electron transport and catalytic activity. Additionally, all of the PWNiO composites displayed reduced charge transfer resistance, facilitating rapid electron and OH^−^ ion transport across the electrode interface, which is crucial for achieving efficient OER kinetics. This study provides an effective strategy for the fabrication of high-performance electrocatalysts with improved charge transfer dynamics and enhanced electrochemical efficiency. The findings suggest that PEG-WO_3_-NiO-based materials hold significant promise for practical electrochemical applications, particularly in energy conversion and storage systems.

## Data Availability

The original contributions presented in the study are included in the article/Supplementary Material, further inquiries can be directed to the corresponding author.

## Supplementary Materials

The following supporting information can be downloaded at: https://www.mdpi.com/article/10.3390/polym17091281/s1, Figure S1: (a,b) SEM micrograph images of WO_3_. Figure S2: (a,b) HRTEM images of PWNiO_1.5_ composite. Figure S3: Reduction areas of (a) PWNiO_0.5_ (b) PWNiO_1_, and (c) PWNiO_1.5_ electrocatalysts from their corresponding CV for OER TOF. Figure S4: Cyclic voltammetry analysis of all the electrocatalysts at different scan rate. Figure S5: 2C_dl_ graph of all the electrocatalysts. Table S1: Comparison of our ternary electro catalysts OER performance result with other reported electrocatalysts. References [21,22,23,44,45,46,47,48,49,50,51] are cited in the supplementary materials.
