# Supplementary material for: Rational Designing of NiO Nanoparticles Anchored with PEG-WO3 for Enhanced Water Oxidation Performance
Source: Polymers (Basel). 2025 May 7;17(9):1281. doi: 10.3390/polym17091281 (PMC12074263; doi:10.3390/polym17091281)
Supplement: Supplementary file 1 [file polymers-17-01281-s001.zip › polymers-3586288-supplementary.pdf]

Rational Designing of NiO Nanoparticles Anchored PEG-WO_3 for Enhanced Water Oxidation Performance.

Mrunal Bhosale, Pritam J. Morankar, Rutuja U. Amate and Chan-Wook Jeon *

School of Chemical Engineering, Yeungnam University, 280 Daehak-ro,

Gyeongsan 712-749, Republic of Korea

* *Correspondence: cwjeon@ynu.ac.kr*

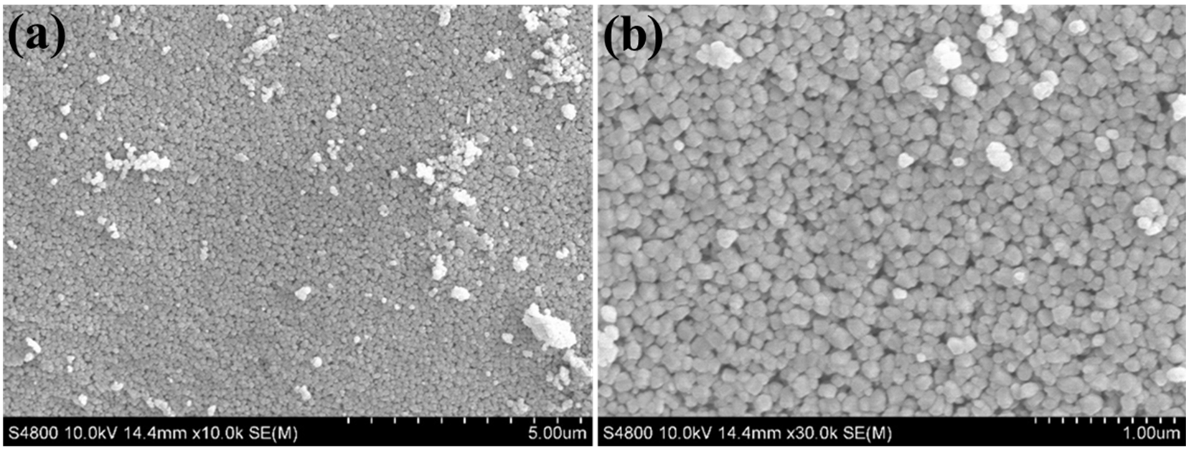

Figure S1: (a,b) SEM micrograph images of WO_3.

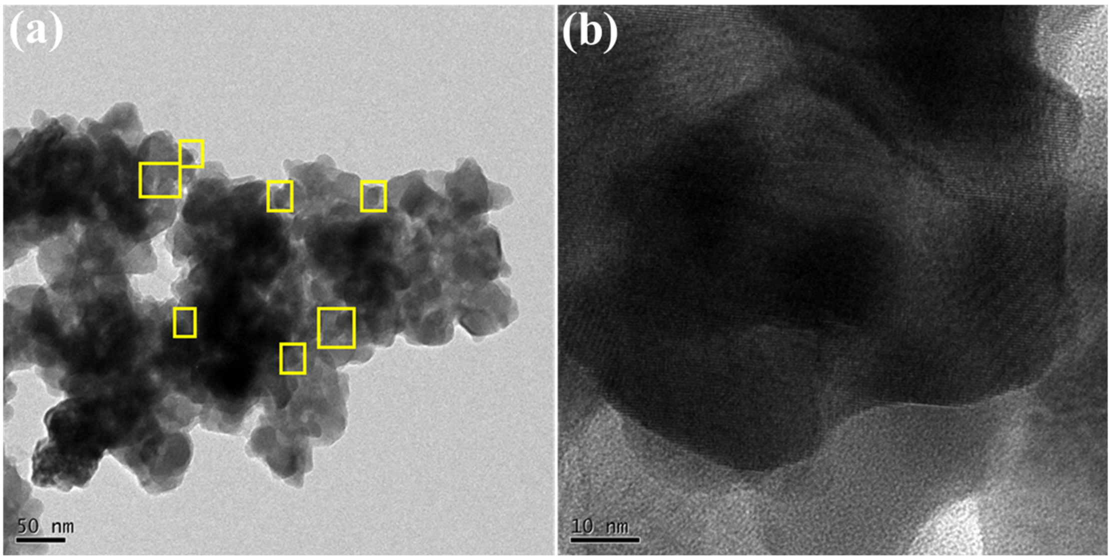

Figure S2: (a,b) HRTEM images of $PWNiO_{1.5}$ composite.

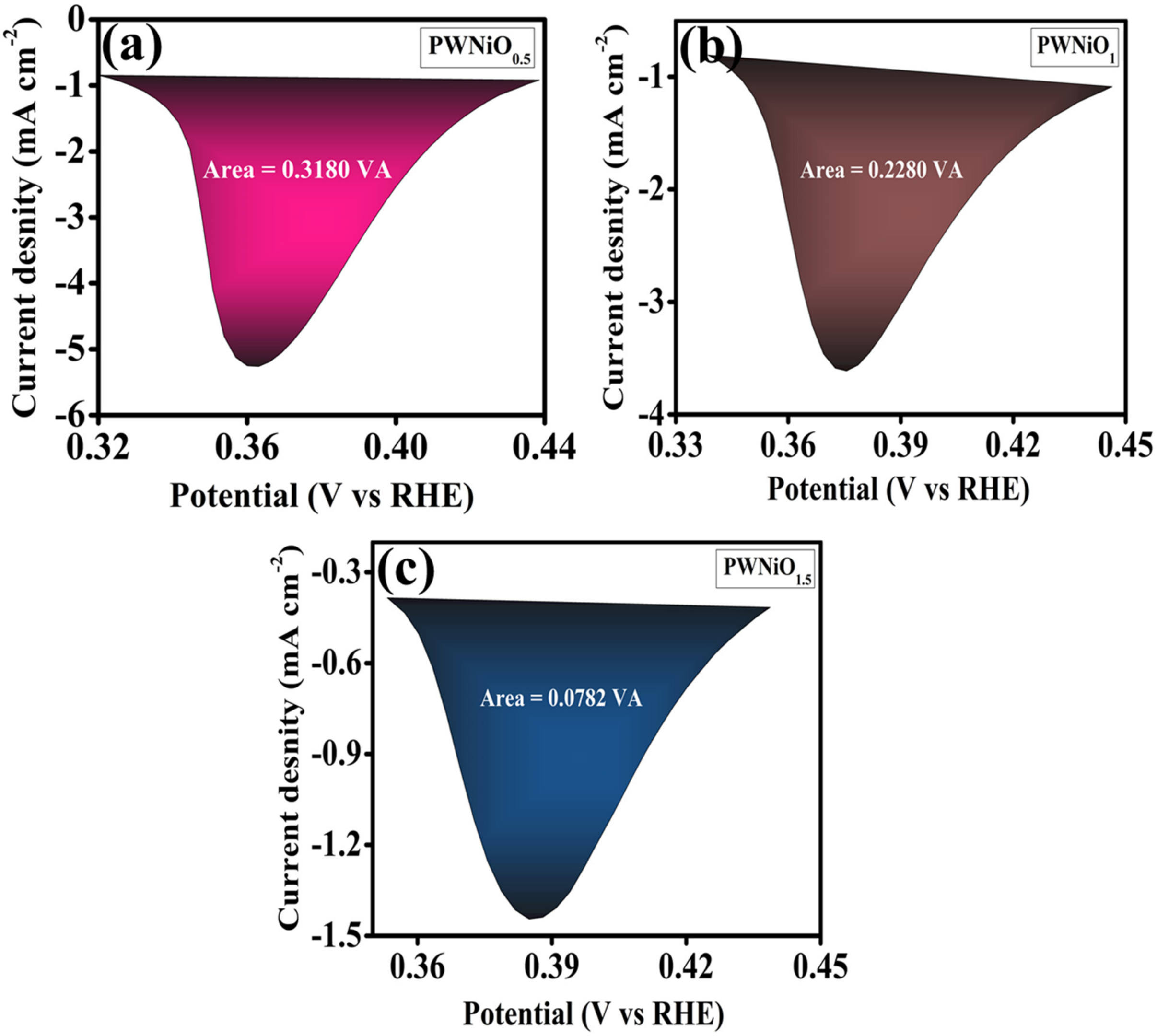

Figure S3: Reduction areas of (a) $PWNiO_{0.5}$ (b) $PWNiO_1$, and (c) $PWNiO_{1.5}$ electrocatalysts from their corresponding CV for OER TOF.

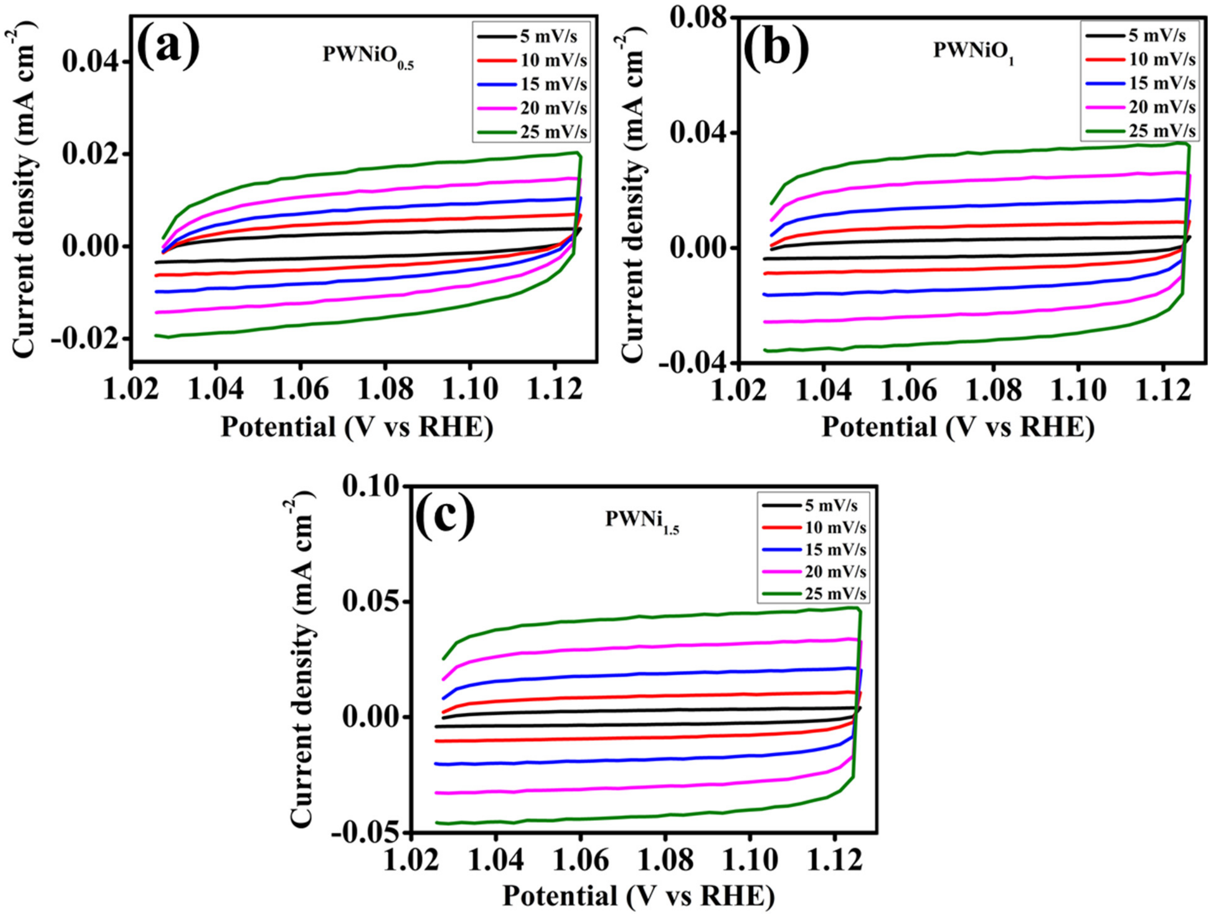

Figure S4: Cyclic voltammetry analysis of all the electrocatalysts at different scan rate.

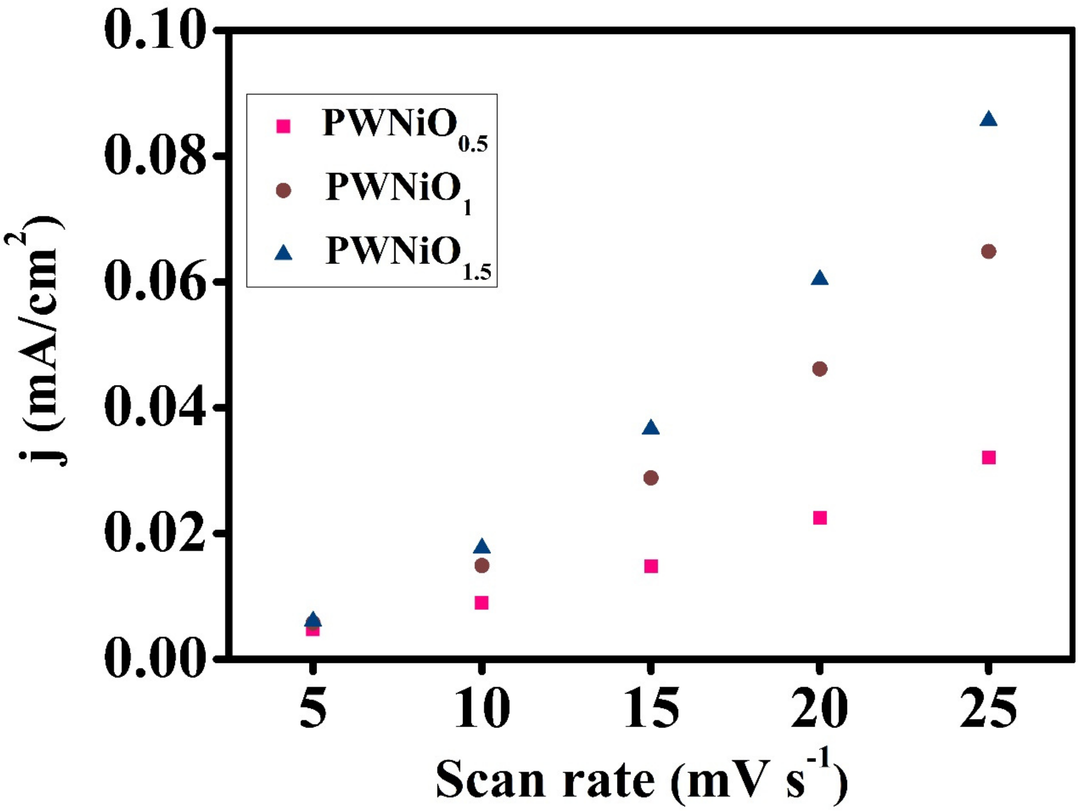

Figure S5: $2C_{dl}$ graph of all the electrocatalysts.

Table S1. Comparison of our ternary electro catalysts OER performance result with other reported electrocatalysts.

**Electrocatalyst**	**Over potential @10 mA cm^{-2} (mV)**	**Tafel Plot (mV dec^{-1})**	**Ref.**
NiO nanoparticles	373	139	[S1]
NiO-300	370	156	[S2]
Ag_2O–NiO	430	98	[S3]
NiO NPs/rGO	369	61	[S4]
carbon layer coated Ni_3S_2/MoS_2	350	79	[S5]
CeO_2/Ni/NC	390	123	[S6]
Co_3Fe_7@Fe_2N/rGO	371	114	[S7]
$Ni(OH)_2$/GO	380	89.1	[S8]
CoTp 3:1(Ni)	371	53.6	[S9]
Ni-MOF@CNT	370	138.2	[S10]
PO-Ni/Ni-N-CNFs	420	113.10	[S11]
$PWNiO_{1.5}$	349.7	71.2	[Present work]

References:

S1. Manjunath, V., Bimli, S., Biswas, R., Didwal, P.N., Haldar, K.K., Mahajan, M., Deshpande, N.G., Bhobe, P.A. and Devan, R.S., 2022. Experimental investigations on morphology controlled bifunctional NiO nano-electrocatalysts for oxygen and hydrogen evolution. International Journal of Hydrogen Energy, 47(92), pp.39018-39029.

S2. Mondal, A., Paul, A., Srivastava, D.N. and Panda, A.B., 2018. NiO hollow microspheres as efficient bifunctional electrocatalysts for overall water-splitting. international journal of hydrogen energy, 43(47), pp.21665-21674.

S3. Shaghaghi, Z. and Akbari, S., 2024. Hydrogen and oxygen production on Ag2O/NiO hybrid nanostructures via electrochemical water splitting. International Journal of Hydrogen Energy, 51, pp.936-949.

S4. Jo, S.G., Kim, C.S., Kim, S.J. and Lee, J.W., 2021. Phase-controlled NiO nanoparticles on reduced graphene oxide as electrocatalysts for overall water splitting. Nanomaterials, 11(12), p.3379.

S5. Wang, C.P., Kong, L.J., Sun, H., Zhong, M., Cui, H.J., Zhang, Y.H., Wang, D.H., Zhu, J. and Bu, X.H., 2019. Carbon layer coated Ni3S2/MoS2 nanohybrids as efficient bifunctional electrocatalysts for overall water splitting. ChemElectroChem, 6(22), pp.5603-5609.

S6. Tian, L., Liu, H., Zhang, B., Liu, Y., Lv, S., Pang, L. and Li, J., 2021. Ni and CeO2 nanoparticles anchored on cicada-wing-like nitrogen-doped porous carbon as bifunctional catalysts for water splitting. ACS Applied Nano Materials, 5(1), pp.1252-1262.

S7. Liang, Dong, et al. "MOFs-derived core-shell Co3Fe7@ Fe2N nanopaticles supported on rGO as high-performance bifunctional electrocatalyst for oxygen reduction and oxygen evolution reactions." Materials Today Energy 17 (2020): 100433.

S8. Upadhyay, S., Mir, R.A. and Pandey, O.P., 2023. Ni (OH) 2 nanosheets and highly stable Ni (OH) 2/GO nanocomposite for its improved OER performance. International Journal of Hydrogen Energy, 48(94), pp.36687-36693.

S9. Sprengel, S., Amiri, M., Bezaatpour, A., Nouhi, S., Baues, S., Wittstock, G. and Wark, M., 2022. One-Pot Synthesis of Ni-MOF/Co-MOF Hybrid as Electrocatalyst for Oxygen Evolution Reaction. Journal of The Electrochemical Society, 169(12), p.124504.

S10. Sreekanth, T.V.M., Dillip, G.R., Nagajyothi, P.C., Yoo, K. and Kim, J., 2021. Integration of Marigold 3D flower-like Ni-MOF self-assembled on MWCNTs via microwave irradiation for high-performance electrocatalytic alcohol oxidation and oxygen evolution reactions. Applied Catalysis B: Environmental, 285, p.119793.

S11. Wu, Z.Y., Ji, W.B., Hu, B.C., Liang, H.W., Xu, X.X., Yu, Z.L., Li, B.Y. and Yu, S.H., 2018. Partially oxidized Ni nanoparticles supported on Ni-N co-doped carbon nanofibers as bifunctional electrocatalysts for overall water splitting. Nano Energy, 51, pp.286-293.
